# Supplementary material for: Metabolic costs and trade-offs of hypermetabolism in human motor neurons with ATP synthase deficiency
Source: Commun Biol. 2025 Dec 11;8:1759. doi: 10.1038/s42003-025-09149-7 (PMC12698834; doi:10.1038/s42003-025-09149-7)
Supplement: Supplementary file 2 — Suplementary information file [file 42003_2025_9149_MOESM2_ESM.pdf]

SUPPLEMENTARY MATERIAL

List of content

- Supplementary Figure 1. ATP synthase deficiency leads to hypermetabolism but does not affect mitochondrial content.
- Supplementary Figure 2. Metabolic tracing over 48 hours reveals the most significant changes.
- Supplementary Figure 3. Mutant iPSC-MN have reduced level of pantothenate, but do not show changes in lactate consumption and do not respond to avanafil treatment.
- Supplementary Figure 4. Most of respiratory complex IV subunits are upregulated, which aligns with a reorganization of the mitochondrial compartment.
- Supplementary Figure 5. Coenzyme A metabolism and part of its fates are not affected in mutant iPSC-MN.
- Supplementary Figure 6. Acetyl-CoA oxidation in mitochondria is not altered in mutant iPSC-MN.
- Supplementary Figure 7. Acetylcholine neurotransmitter levels are not affected in mutant iPSC-MN.
- Supplementary Figure 8. N-terminal acetylases are not affected in mutant iPSC-MN.
- Supplementary Figure 9: Uncropped and unedited blot/gel images
- Supplementary Material Bibliography

Supplementary Figure 1

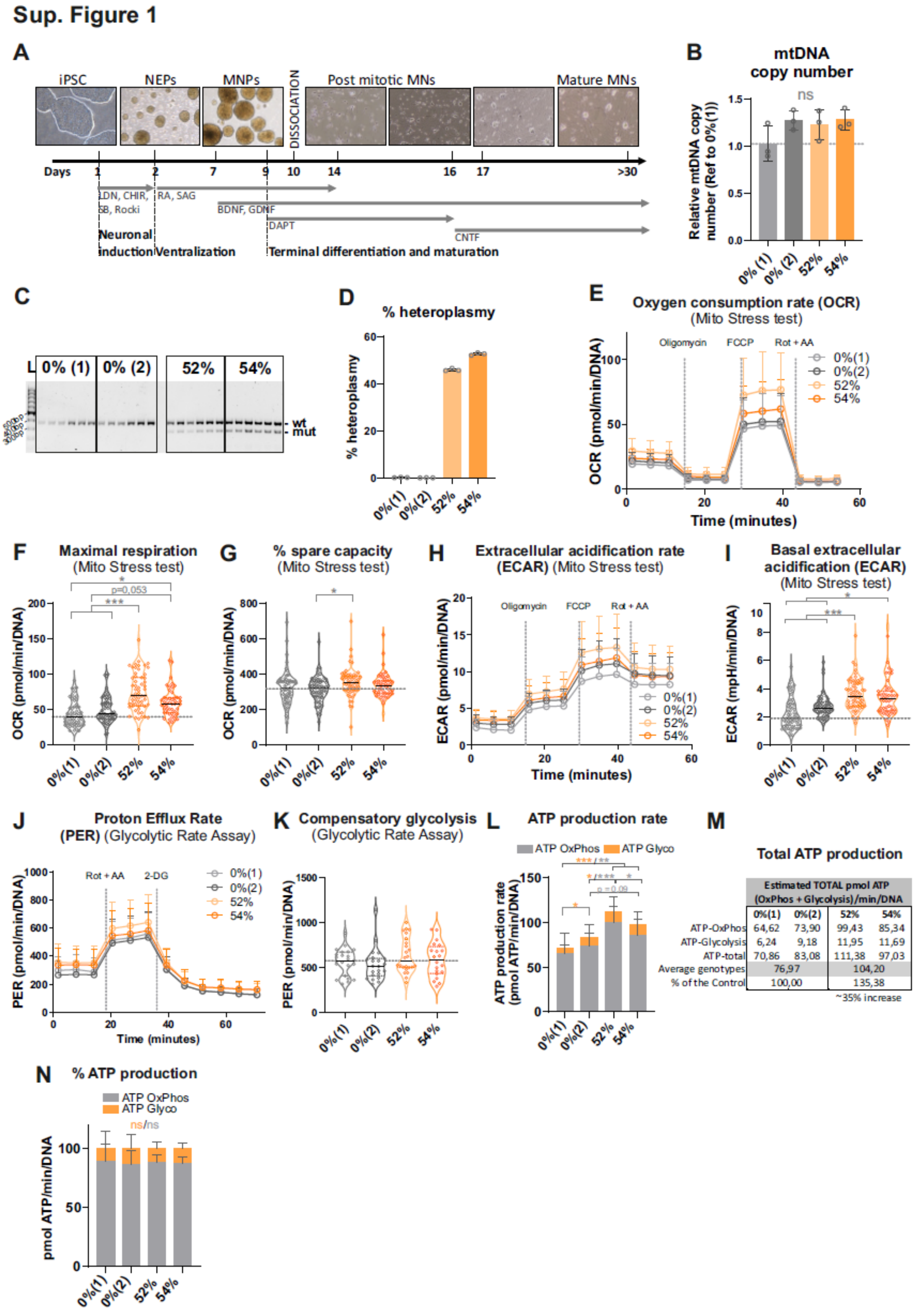

	Estimated TOTAL pmol ATP (OxPhos + Glycolysis)/min/DNA			
	0%(1)	0%(2)	52%	54%
ATP-OxPhos	64,62	73,90	99,43	85,34
ATP-Glycolysis	6,24	9,18	11,95	11,69
ATP-total	70,86	83,08	111,38	97,03
Average genotypes	76,97		104,20	
% of the Control	100,00		135,38	

~35% increase

Supplementary Figure 1. ATP synthase deficiency leads to hypermetabolism but does not affect mitochondrial content. (A) Motor neuron differentiation protocol scheme depicting the timing of supplementation with small molecules. Based on [1,2]. **(B)** Mitochondrial DNA copy number on day 30 measured by qPCR. The data represents n=3 independent differentiation experiments, each with three independent wells measured by duplicate. (**C)** Representative image of an agarose gel depicting the RFLP analysis from iPSC-MN day 30 including digested samples with HpyF31. Upon digestion, an additional band can be observed only in the mutant clones (mut). **(D)** Quantification of **(C)** as the fraction of mutant peak intensity / total. Each dot represents an independent motor neuron differentiation (n_exp = 3 independent differentiations). For each differentiation, heteroplasmy levels were measured from two separate wells (n_technical_replicates = 2), with each measurement performed in duplicate. The average of the two technical replicates was used to generate a single data point for each differentiation. **(E-I)** Extended Seahorse XF Mito Stress test results from **Figure 1**, n = 13-21 wells per cell line per experiment, from three independent differentiations. **(E)** Mitochondrial oxygen consumption rate (OCR) kinetic traces depicting three basal respiration rate measurements followed by another three measurements after each injection (oligomycin, FCCP and Rotenone + antimycin). **(F)** Maximal respiration calculated after FCCP injection to induce uncoupling. **(G)** % spare capacity was calculated as the difference between basal respiration and maximal respiration and expressed as %. **(H)** Extracellular acidification rate (ECAR) curves obtained simultaneously as in **(E)**. **(I)** Basal ECAR measured before injecting oligomycin and extracted from (H). **(J-K)** Extended Seahorse XF Glycolytic Rate Assay results from **Figure 1.** n = 9-11 wells per cell line per experiment, from two independent Seahorse experiments. **(J)** Kinetic curves of proton efflux rate (PER). **(K)** Compensatory glycolysis, calculated after rotenone and antimycin injection and post-2-DG acidification. **(L-N)** ATP production rates derived from the Seahorse XF Mito stress test results and calculated based on [3]. **(L)** ATP production through OxPhos and glycolysis, shown separately using the same data as in Figure 1J. **(M)** Computation of % of hypermetabolism in mutant iPSC-MN based on total ATP production (ATP-OxPhos + ATP-glycolysis) derived from Figure 1J. **(N)** % total ATP production derived from **(L)** to determine difference in pathway preference. Data shown as mean ± standard deviation, *$p<0.05$, **$p<0.01$, ***$p<0.001$, or *ns* = non-significant by One-Way ANOVA followed by Bonferroni´s (Seahorse) or Tukey´s multiple comparison post-hoc test.

Supplementary Figure 2

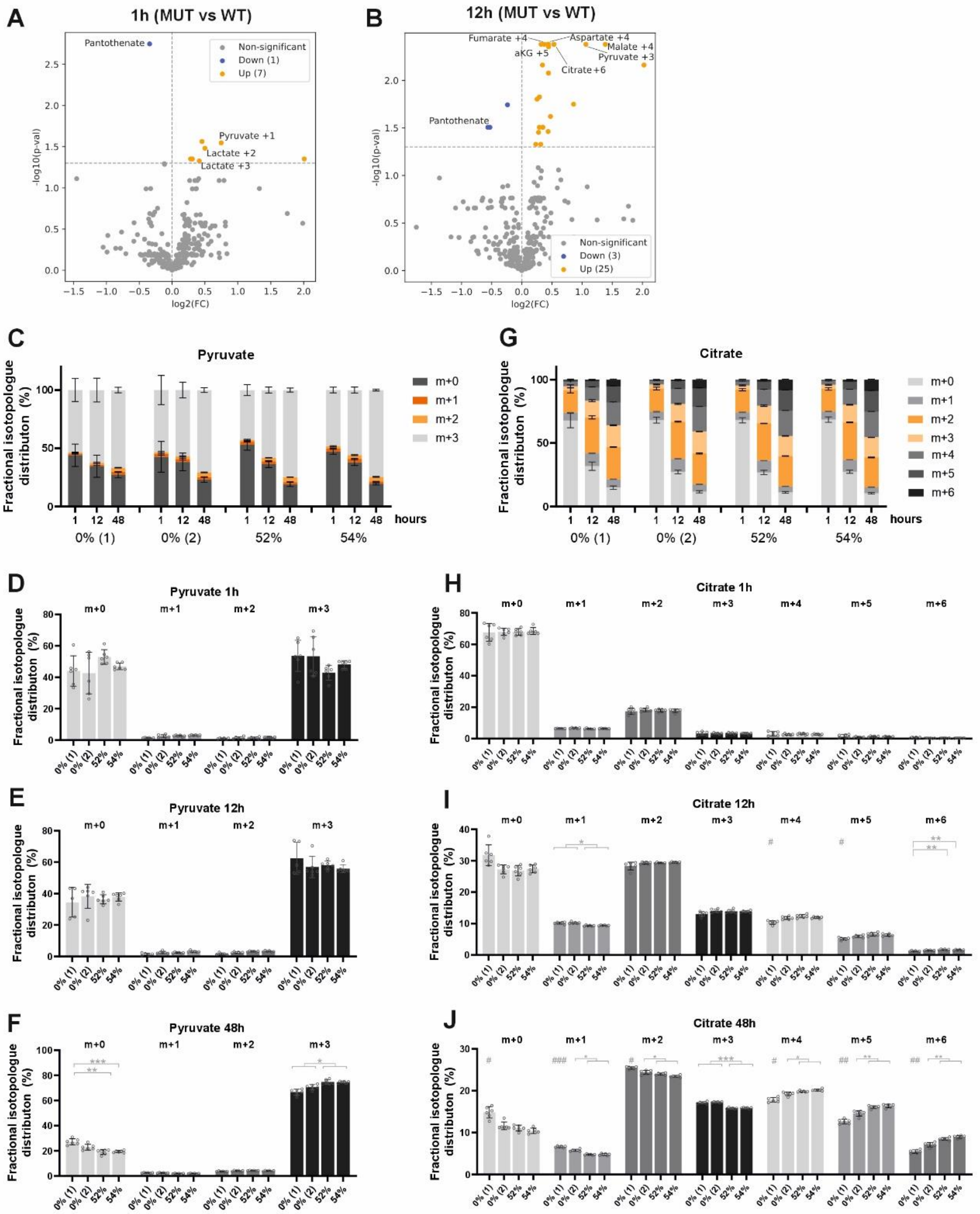

Supplementary Figure 2. Metabolic tracing over 48 hours reveals the most significant changes. **(A-B)** Volcano plots showing significant changes in metabolites after 1 hour **(A)** and 12 hours **(B),** as in **Fig. 2c**. **(C)** Fractional distribution analysis of pyruvate isotopologues (m+0 to m+3), where the sum of all represents 100%, changes over time. With longer labeling time, the proportion of labeled fractions (m+3) increases. **(D)** Fractional distribution analysis of pyruvate isotopologues separated by labeling times (1, 12, or 48 hours) to facilitate the visualization of significant differences in isotopic labeling over time. **(E)** Fractional

distribution analysis of citrate isotopologues (m+0 to m+6), where the sum of all represents 100%, changes over time. With longer labeling time, the proportion of labeled fractions (m+2 to m+6) increases. **(F)** Fractional distribution analysis of citrate isotopologues separated by labeling times (1, 12, or 48 hours) to facilitate the visualization of significant differences in isotopic labeling over time. Most clear changes appear after 48 hours, so we focused on this time point for the analysis. Data shown as mean ± standard deviation, **p*<0.05, ***p*<0.01, ****p<0.001*, # vs all, by One-Way ANOVA followed by Tukey´s multiple comparison post-hoc test.

Supplementary Figure 3

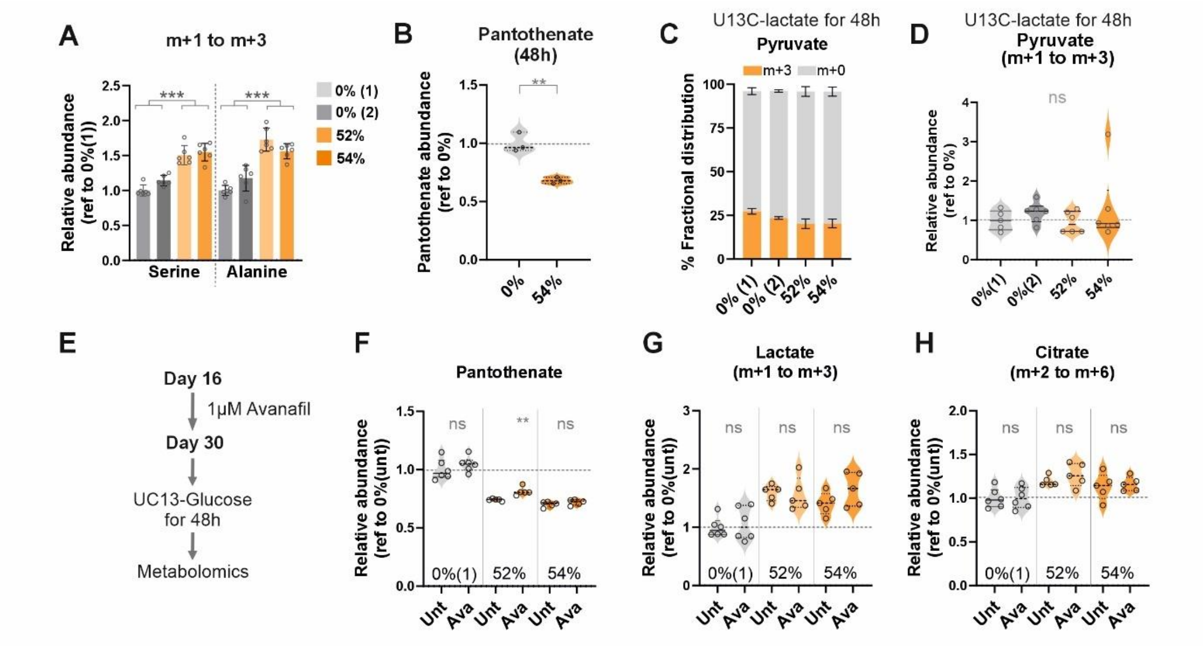

Supplementary Figure 3. Mutant iPSC-MN have reduced level of pantothenate, but do not show changes in lactate consumption and do not respond to avanafil treatment. (A) Relative abundance of serine and alanine after 48 hours of labeling with fully labeled glucose. **(B)** Relative abundance of pantothenate from an independent differentiation after 48 hours labeling with uniformly 13C-labelled glucose to validate the initial results. **(C-D)** 48 hours of labeling with fully labeled lactate (U-13C) in media containing non-labeled glucose. **(C)** Fractional distribution analysis of pyruvate isotopologues (m+0 and m+3). iPSC-MN were able to metabolize lactate back into pyruvate, as evidenced by the presence of approximately 20-25% labeled pyruvate. **(D)** Relative abundance of labeled pyruvate. **(E-H)** Avanafil (1 µM) was added to the culture medium every other day from day 16 to day 30. On day 30, cells were switched to medium containing uniformly 13C-labelled glucose, and metabolic labelling proceeded for 48 hours in the presence of Avanafil. **(F)** Pantothenate levels in Unt (untreated) and Ava (Avanafil) treated cells. **(G)** Labeled lactate (m+1 to m+3) levels in Unt (untreated) and Ava (Avanafil) treated cells. **(H)** Labeled citrate (m+2 to m+6) levels in Unt (untreated) and Ava (Avanafil) treated cells. Data shown as mean ± standard deviation, **$p<0.01$, ***$p<0.001$, ns = non-significant, by One-Way ANOVA followed by Tukey´s multiple comparison post-hoc test or t-test (Avanafil treatments, Unt vs Ava).

Supplementary Figure 4

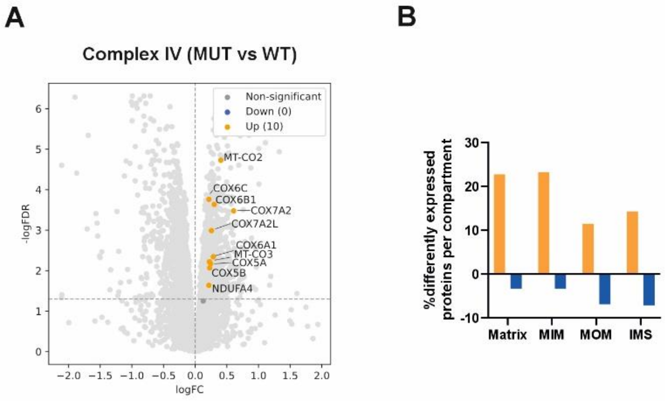

Supplementary Figure 4. Most of respiratory complex IV subunits are upregulated, which aligns with a reorganization of the mitochondrial compartment. (A) Volcano plot depicting protein expression changes of the complex IV subunits between MUT and WT Thresholds: FDR = 0.01, log2(FC) = 1. **(B)** Percentage of differently expressed proteins per mitochondrial compartment: matrix (total: 453, down: 15, up 103), mitochondrial inner membrane (MIM, total: 269, down: 7, up: 63), mitochondrial outer membrane (MOM, total: 87, down: 6, up: 10) and mitochondrial intermembrane space (IMS, total: 42, down: 3, up: 6).

Supplementary Figure 5

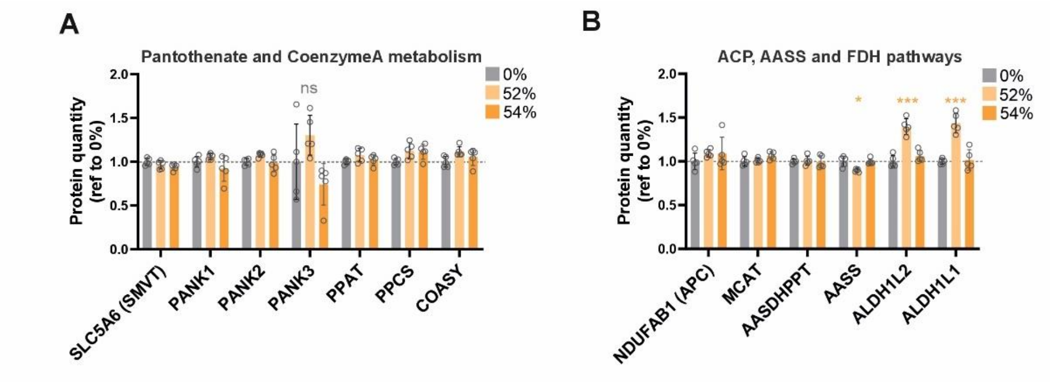

Supplementary Figure 5. Coenzyme A metabolism and part of its fates are not affected in mutant iPSC-MN. **(a)** Protein expression levels extracted from the proteomics data set for the pantothenate transporter on the plasma membrane (SMVT) and the enzymes involved in its conversion into coenzyme A. **(b)** Protein expression levels extracted from the proteomics data set for the acyl protein carrier pathway (ACP and MCAT), α-aminoadipate semialdehyde synthase pathway (AASDHPPT and AASS) and folate cycle (ALDH1L1 and 2). Data shown as mean ± standard deviation, **p<0.05*, ****p<0.001*, by One-Way ANOVA followed by Tukey´s multiple comparison post-hoc test.

Supplementary Figure 6

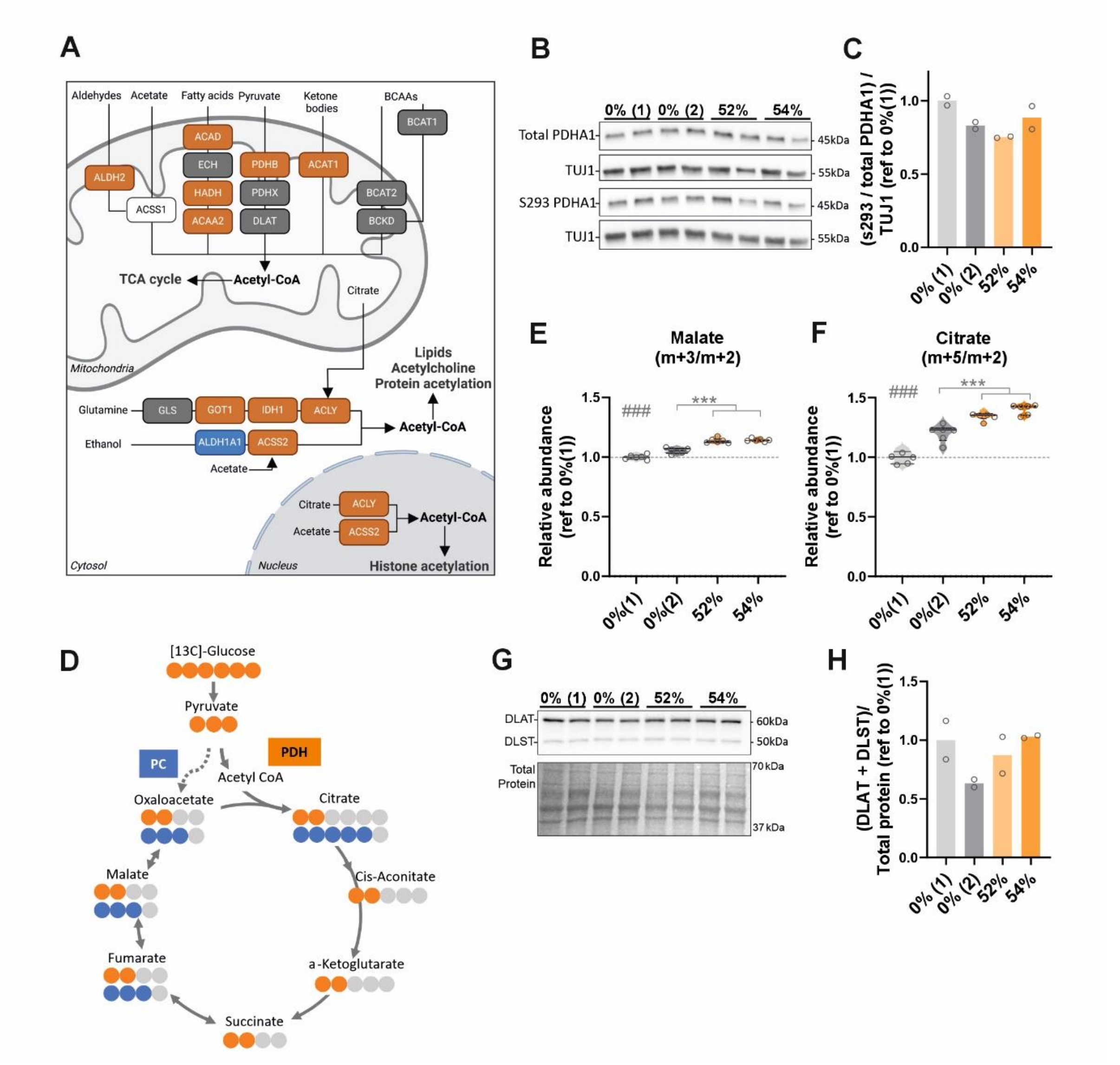

Supplementary Figure 6. Acetyl-CoA oxidation in mitochondria is not altered in mutant iPSC-MN. **(A)** Scheme showing proteins involved in the production of acetyl-CoA from various sources across different cellular compartments. Based on [4,5]. Orange denotes upregulation, blue indicates downregulation, grey indicates no changes and white signifies that the protein was not found in the dataset. BCAAs, branched-chain amino acids; FAS, fatty acid synthesis. Created in BioRender. Torregrosa, R. (2025) https://BioRender.com/k97l865. **(B)** Representative cropped immunoblot of total PDHA and the inactivating phosphorylation at S293. **(C)** Quantification of **(B).** Total and S293 PDHA were normalized to TUJ1 (loading control), and then the ratio S293/total was calculated. **(D)** Pyruvate can enter the TCA cycle through two different pathways. Pyruvate carboxylation catalyzed by pyruvate carboxylase (PC, shown in blue) transfers all three carbons from pyruvate into oxaloacetate. In contrast, the

second pathway involves the pyruvate dehydrogenase complex (PDH, shown in orange), which transfers only two carbons from pyruvate (labeled as m+2) into citrate. This results in a citrate molecule containing five labeled carbons (m+5), composed of two carbons from PDH and three carbons from the previous cycle of PC activity. **(E and F)** Representative ratios to determine PC or PDH activity preference after 48h of isotopic labeling. Increased ratios denote elevated PC activity. Malate represents the entry point for the PC pathway, as oxaloacetate could not be detected, while citrate does for the PDH pathway. **(G)** Representative cropped immunoblot of lipoylated proteins DLAT and DSLT detected with an anti-lipoic acid antibody. **(H)** Quantification of **(G)**. The intensity of DLAT + DSLT was normalized to total protein (Poncaeu staining).

Supplementary Figure 7

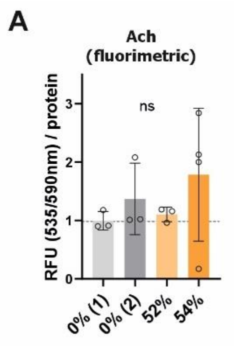

Supplementary Figure 7. Acetylcholine neurotransmitter levels are not affected in mutant iPSC-MN. (A) Acetylcholine levels measured in total cellular lysates using a fluorometric kit from day 36 motor neurons. n=3-4 technical replicates (individual wells) measured by duplicate. *ns* = non-significant.

Supplementary Figure 8

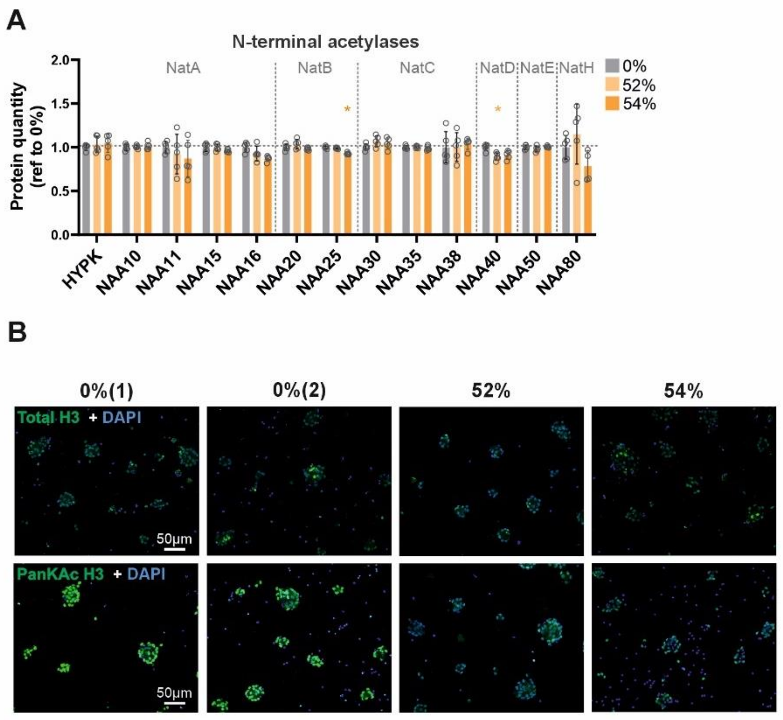

Supplementary Figure 8. N-terminal acetylases are not affected in mutant iPSC-MN. (A) Protein expression levels extracted from the proteomics data set for the family of N-terminal acetylases enzymes (NATs). NatD is also involved in histone acetylation [6]. (**B**) iPSC-derived motor neurons on day 30. Upper panels, representative immunocytochemistry images of total histone H3 (green) and nucleus (Dapi, blue). Scale bar 50µm. Lower panels, representative immunocytochemistry images of total PankAc H3 (K9 + K14 + K18 + K23 + K27) (green) and nucleus (Dapi, blue). Scale bar 50µm.

Supplementary Figure 9

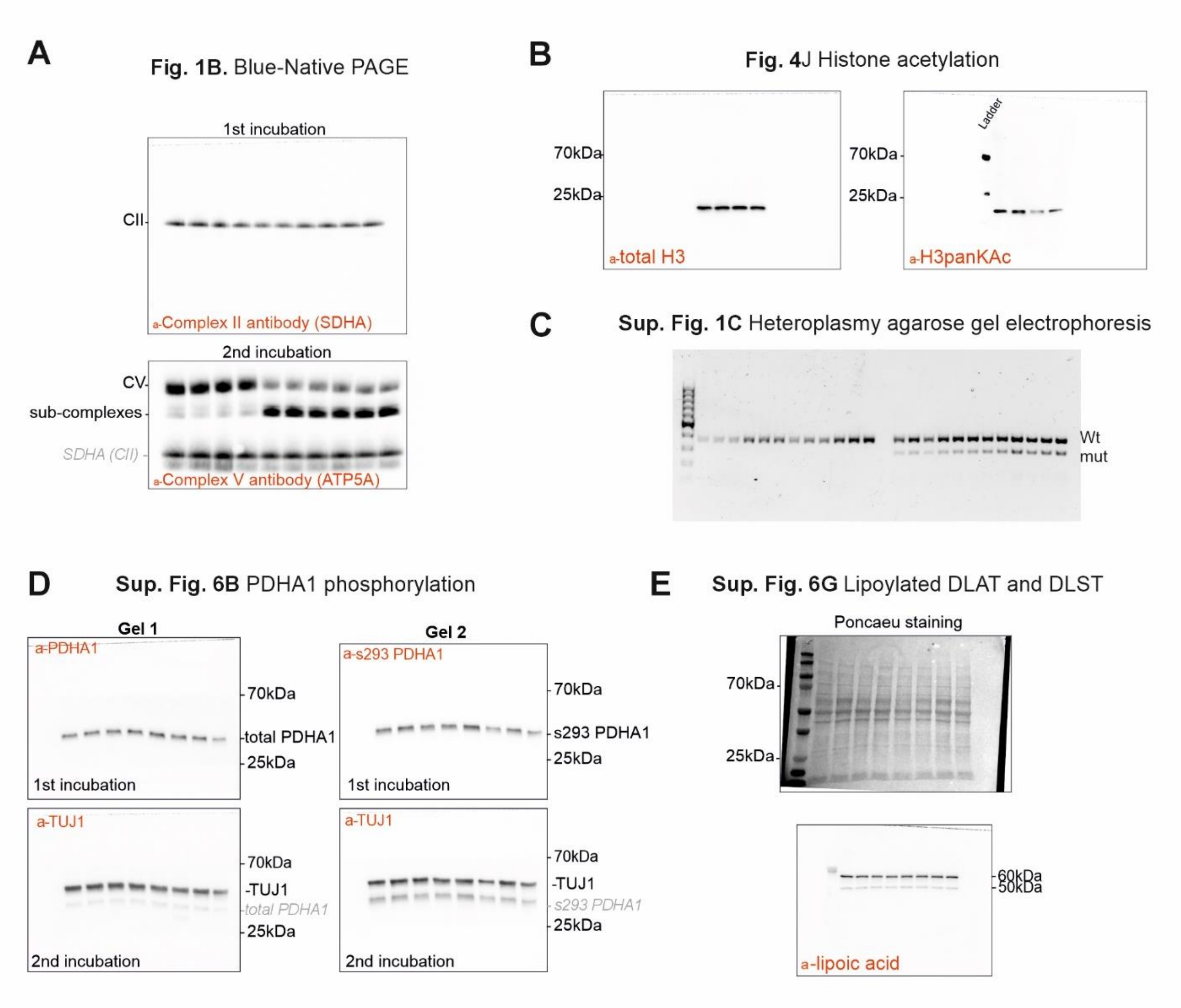

Supplementary Figure 9. Uncropped and unedited blot/gel images. (A) *Figure 1B*, Blue Native Page. **(B)** *Figure 4J* Histone acetylation. **(C)** *Sup. Figure 1C* Heteroplasmy agarose gel electrophoresis. **(D)** *Sup. Figure 6B* PDHA1 phosphorylation. **(E)** *Sup. Figure 6G* Lipoylated DLAT and DLST.

Supplementary Material Bibliography

1 Guo, W., Naujock, M., Fumagalli, L. *et al.* HDAC6 inhibition reverses axonal transport defects in motor neurons derived from FUS-ALS patients. *Nat Commun* **8**, 861 (2017).

2 Maury, Y., Come, J., Piskorowski, R. A. *et al.* Combinatorial analysis of developmental cues efficiently converts human pluripotent stem cells into multiple neuronal subtypes. *Nat Biotechnol* **33**, 89-96 (2015).

3 Desousa, B. R., Kim, K. K., Jones, A. E. *et al.* Calculation of ATP production rates using the Seahorse XF Analyzer. *EMBO Rep* **24**, e56380 (2023).

4 Pietrocola, F., Galluzzi, L., Bravo-San Pedro, J. M., Madeo, F. & Kroemer, G. Acetyl coenzyme A: a central metabolite and second messenger. *Cell Metab* **21**, 805-821 (2015).

5 Bradshaw, P. C. Acetyl-CoA Metabolism and Histone Acetylation in the Regulation of Aging and Lifespan. *Antioxidants (Basel)* **10** (2021).

6 Aksnes, H., McTiernan, N. & Arnesen, T. NATs at a glance. *J Cell Sci* **136** (2023).
