# Supplementary material for: Metabolic costs and trade-offs of hypermetabolism in human motor neurons with ATP synthase deficiency
Source: Commun Biol. 2025 Dec 11;8:1759. doi: 10.1038/s42003-025-09149-7 (PMC12698834; doi:10.1038/s42003-025-09149-7)
Supplement: Supplementary file 3 — Description of Additional Supplementary Files [file 42003_2025_9149_MOESM3_ESM.pdf]

Description of Additional Supplementary Files

File name: Supplementary Data 1

Description: metabolomics data, including raw and normalized values, relative abundances, fractional distributions, ratios, and pairwise comparisons.

File name: Supplementary Data 2

Description: proteomics data, including protein abundance and unique peptide counts for each cell line or genotype, as well as pathway enrichment analysis results.

File name: Supplementary Data 3

Description: source data for all figures.
